# Post-TB care in the UK: a national survey of existing practice

**DOI:** 10.1136/bmjresp-2025-004021

**Published:** 2026-02-26

**Authors:** Ailva O’Reilly, Christopher Andrew Martin, Sharon Elizabeth Cox, Pranabashis Haldar, Dominik Zenner, Manish Pareek, Jamilah Meghji

**Affiliations:** 1 Division of Public Health and Epidemiology, School of Medical Sciences, University of Leicester College of Life Sciences, Leicester, UK; 2 Development Centre for Population Health, University of Leicester, Leicester, UK; 3 NIHR Leicester Biomedical Research Centre (BRC), University of Leicester, Leicester, UK; 4 Department of Infection and HIV Medicine, University Hospitals of Leicester NHS Trust, Leicester, UK; 5 TB Unit, UKHSA, London, UK; 6 Faculty of Epidemiology and Population Health, London School of Hygiene & Tropical Medicine, London, UK; 7 Division of Respiratory Sciences, School of Medical Sciences, University of Leicester College of Life Sciences, Leicester, UK; 8 NIHR Leicester Biomedical Research Centre, Leicester, UK; 9 Global Public Health Unit, Wolfson Institute of Population Health, Queen Mary University, London, UK; 10 Queen Mary and Barts Health TB Centre, Blizard Institute, Queen Mary University London, London, UK; 11 National Heart and Lung Institute, Imperial College London, London, UK

**Keywords:** Tuberculosis, Surveys and Questionnaires

## Abstract

**Background:**

Tuberculosis (TB) survivors experience high mortality and long-term morbidity, contributing substantially to the global TB burden. In the UK, where TB incidence is rising, the scale of post-TB health needs is unknown and current guidelines do not recommend follow-up. We conducted the first nationwide survey of UK TB services to assess approaches to post-TB care.

**Methods:**

We conducted a digital survey between February and May 2025 across National Health Service TB services in all four nations, targeting specialist clinicians. The questionnaire captured data on types of post-TB morbidity encountered and current practice. We analysed descriptively and stratified by caseload.

**Results:**

We received responses from 113 of 135 TB services (84%). Most respondents were lead clinicians (81%), and nearly all (96%) had encountered post-TB morbidity in their patient populations, including lung disease (82%), social vulnerabilities (79%), and financial issues (66%). High caseload services (≥30 cases/year) reported more types of morbidity (mean 4.2 vs 2.9; p<0.001). While end of treatment symptom screening and chest X-rays are routine (>95%), fewer than half of services perform assessments for broader post-TB sequelae and comorbidities, or provide direct ongoing medical care (41%). Most services cited staffing (78%), clinic capacity (70%) and funding (59%) as challenges to post-TB care.

**Conclusions:**

A high proportion of UK TB clinicians recognise post-TB morbidity among their patient groups. TB services are introducing elements of post-TB care, but provision is heterogenous and often informal, with multiple resource-related challenges. Robust UK-specific data, stakeholder engagement and clear guidance are needed to support post-TB care pathways.

**WHAT IS ALREADY KNOWN ON THIS TOPIC**

- Global research shows tuberculosis (TB) survivors face long-term health problems, and many countries are adopting post-TB care guidelines, but the current state of care in the UK is unclear.

**WHAT THIS STUDY ADDS**

- We show that UK TB clinicians recognise long-term physical and psychosocial problems among TB survivors.
- While follow-up care is being offered by some TB services, this is variable and constrained by resources.

**HOW THIS STUDY MIGHT AFFECT RESEARCH, PRACTICE OR POLICY**

- As TB rates in the UK continue to increase, our findings highlight the need for research to better understand the health of TB survivors in the UK, in order to inform approaches to post-TB care in the National Health Service.

## Introduction

Global research has shown that tuberculosis (TB) survivors (TBS), people who have previously experienced active TB disease, have three times higher rates of mortality^1^ compared with the general population. They experience high rates of pulmonary and systemic morbidity, including obstructive and restrictive lung disease,^2^ cardiovascular disease,^3^ cancer^4^ and mental health problems,^5^ and many remain economically vulnerable.^6 7^ Current estimates state that almost half of the global health burden of TB disease (in disability-adjusted life-years) is attributable to post-TB sequelae.^8^ TBS and researchers have therefore called for the provision of care after anti-TB treatment to improve long-term quality of life.^9^,^11^

In the UK, over 170 000 people have been diagnosed with active TB disease since 2000^12^ and incidence rates for TB are rising. The rate in England was 9.37 per 100 000 population in 2024, representing the largest year-on-year increase (12.4%) since 2000.^12^ However, the number of TBS and their burden of chronic disease and disability in the UK today is not known.

TB in the UK disproportionately affects people who were born in high incidence countries, experience deprivation and have specific social risk factors such as drug or alcohol dependency.^12 13^ People from these underserved groups face a high burden of comorbidities related to poverty and poor access to healthcare,^14^ which are potentially compounded by the long-term effects of TB disease. TBS in the UK may therefore experience significant unmet health needs.

Current National Institute for Clinical Excellence (2016) guidance does not recommend routine follow-up after treatment of drug-susceptible TB. However, this assessment was based on the limited cost effectiveness of follow-up for detecting the low rates of TB relapse or recurrence (3.5%–4%) seen in the UK.^15^ It did not consider the broader aspects of health and well-being after TB and is in contrast with emerging guidance from several countries, including low-incidence settings such as Canada, who have recently adopted standards for assessing and managing post-TB morbidity.^16 17^

Given the lack of guidance on care for TBS in the UK, and data on their potential unmet needs, we undertook the first nationwide survey to evaluate current approaches to post-TB care among National Health Service (NHS) based TB services across all four nations.

## Materials and methods

We report this study as per the Checklist for Reporting Results of Internet E-Surveys (online supplemental checklist S1).^18^

### Study questionnaire

We developed and piloted an 11-point digital questionnaire among specialist doctors and nurses working in TB clinics (online supplemental questionnaire S1). The questionnaire was prefaced by definitions for TBS, post-TB morbidity and post-TB care, to ensure clarity of these concepts. We collected data on types of post-TB morbidity encountered by TB clinicians; the assessments, investigations and referrals usually performed at the end of TB treatment; and whether any post-TB care is currently being provided or planned by the service. For most questionnaire items, respondents were asked to either select from a list of options provided (including ‘other’ and ‘none’), or to select from the options ‘Yes,’ ‘No’ or ‘Not sure.’ For some questions, respondents could enter free text answers.

We also collected descriptive data on whether a respondent was the Lead Consultant or Lead Nurse for their service, their specialty and their TB facility’s caseload.

#### Survey frame

The survey frame included TB services providing adult care within NHS Trusts (England), University Health Boards (Wales), Health and Social Care Trusts (Northern Ireland) and Scottish Health Boards. It was based on primary lists of TB services obtained from key stakeholders in each of the four nations (Acknowledgements), which we refined with input from regional representatives and study participants.

The final survey frame included 135 TB services, including 110 in England, 6 in Wales, 13 in Scotland and 6 in Northern Ireland.

#### Survey distribution and administration

We distributed surveys by email between February and May 2025. For England, Wales and Northern Ireland, the survey was sent directly to at least one specialist consultant or nurse per TB service. For Scotland, Public Health Scotland administrators disseminated the survey directly to the Scottish TB Network mailing list.

We gave presentations in regional and national TB meetings (n=12) to encourage participation. Up to three reminder emails and a phone call were used to follow up with services not responding to the initial email invitation.

Survey completion was voluntary and no incentives were offered for participation. All respondents provided written informed electronic consent.

We collected survey data in the RedCAP (Research Electronic Data Capture) survey platform.^19^ One response was analysed per TB service; where multiple responses were submitted, we selected the first response from the most senior clinician (online supplemental full methods S1).

#### Analysis

We analysed numeric data from questionnaire items descriptively. The median self-reported caseload for TB services was used as a prespecified cut-off (low <median, high ≥median cases/year) for creating a binary caseload variable, by which questionnaire data were stratified. Where caseload was not self-reported, Clinical Commissioning Group-level data for the TB service were substituted (online supplemental full methods S1). We summarised categorical variables using frequency and percentage, and continuous or discrete variables using means (SD) if normally distributed and medians (IQR) for other distributions.

We compared responses in low versus high caseload services using χ² tests and t-tests for categorical and continuous data, respectively. We reported the proportion of missing data for each questionnaire item.

We conducted all numerical analyses using Stata (StataCorp. 2023. Stata Statistical Software: Release 18.0, StataCorp) and identified broad themes from the free text responses.

#### Patient and public involvement

A study-specific patient and public involvement and engagement group, including TBS and specialist TB clinicians, reviewed and refined draft survey questions, advising on wording and acceptability. Their input informed revisions before piloting.

## Results

### Response rates

**Figure 1:**
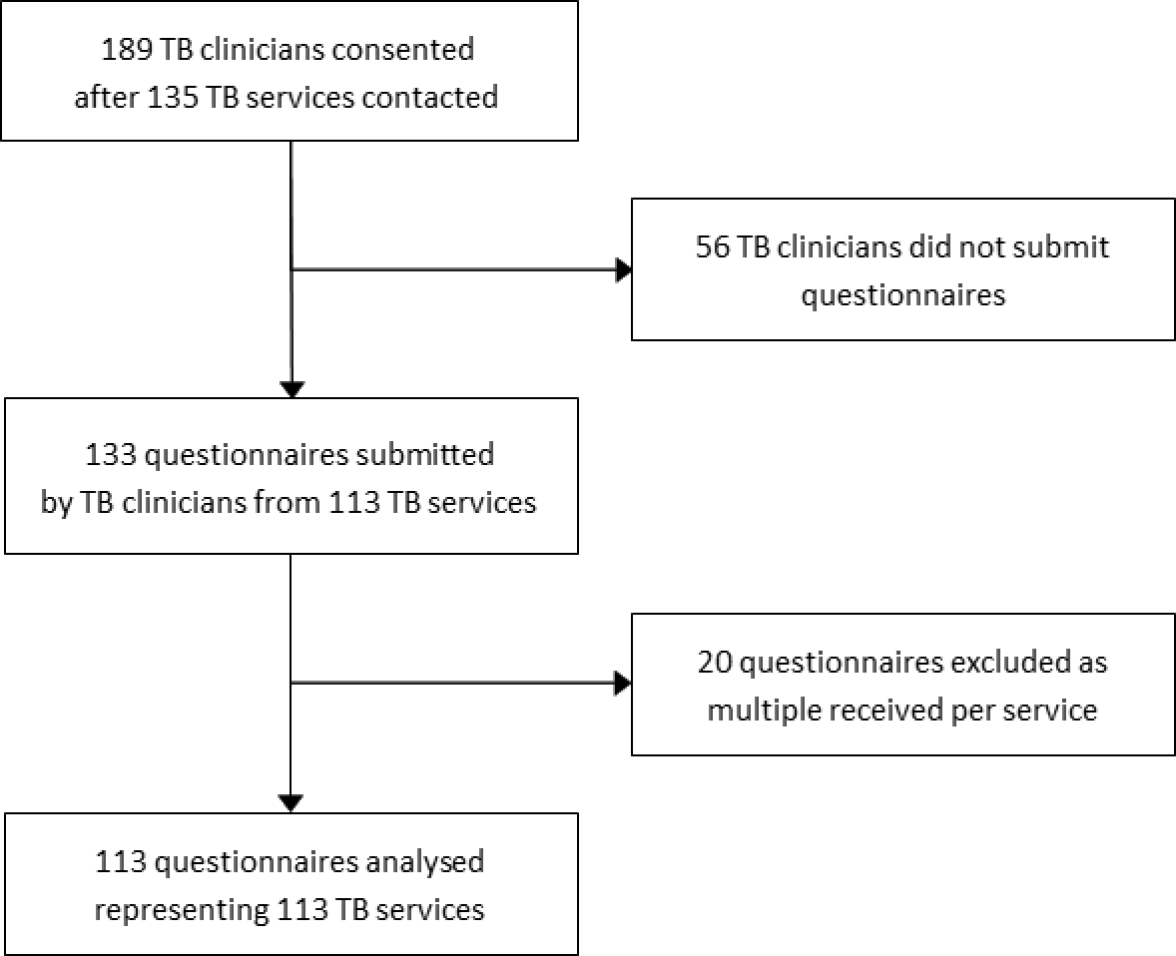
Recruitment and formation of the analysis sample. At least one TB clinician was contacted for each of the 135 TB services in the survey frame and 133 unique questionnaires were submitted. 20 questionnaires were excluded based on the approach described in online supplemental full methods S1. TB, tuberculosis.

Responses were received from 84% (113/135) of TB services (figure 1), with response rates of 85% (94/110) for England, 67% (4/6) for Wales, 77% (10/13) for Scotland and 83% (5/6) for Northern Ireland.

Most questionnaire items (9/11) were completed by all respondents, other than questions 7 and 10, which were 97% and 98% complete, respectively (online supplemental table S2).

### Description of the analysed cohort

Most TB services were represented by a lead clinician (81%; 91/113) including 56 lead nurses (62%), and 35 lead doctors (38%) (online supplemental table S1).

Annual caseload was self-reported (number of active TB cases in 2023) by 110 (97%) of the 113 TB services represented, and obtained from CCG-level data (3-year average annual number of notifications 2021–2023) for the remaining three. The median (self-reported) caseload was 30 (IQR: 15–61) cases of TB per year. 30 was therefore chosen as the cut-off for the binary caseload variable (low <30 cases/year, high ≥30 cases/year) used for stratification.

### Experience of post-TB morbidity

Most survey respondents (96%; 108/113) had come across at least one type of post-TB morbidity among TBS. Among the physical types of post-TB morbidity reported, post-TB lung disease was the most widely recognised (82% of respondents; 93/113), followed by neurological morbidity (41%; 46/113) and post-TB cardiovascular and pericardial disease (18%; 20/113). Many respondents recognised psychosocial morbidity among their patients at or after TB treatment completion, with high proportions having observed problems relating to social vulnerabilities (eg, homelessness, drug or alcohol dependency or forced migrant status) (79%; 89/113) and financial (69%; 78/113) and psychological well-being (66%; 75/113) (online supplemental table S2).

**Figure 2:**
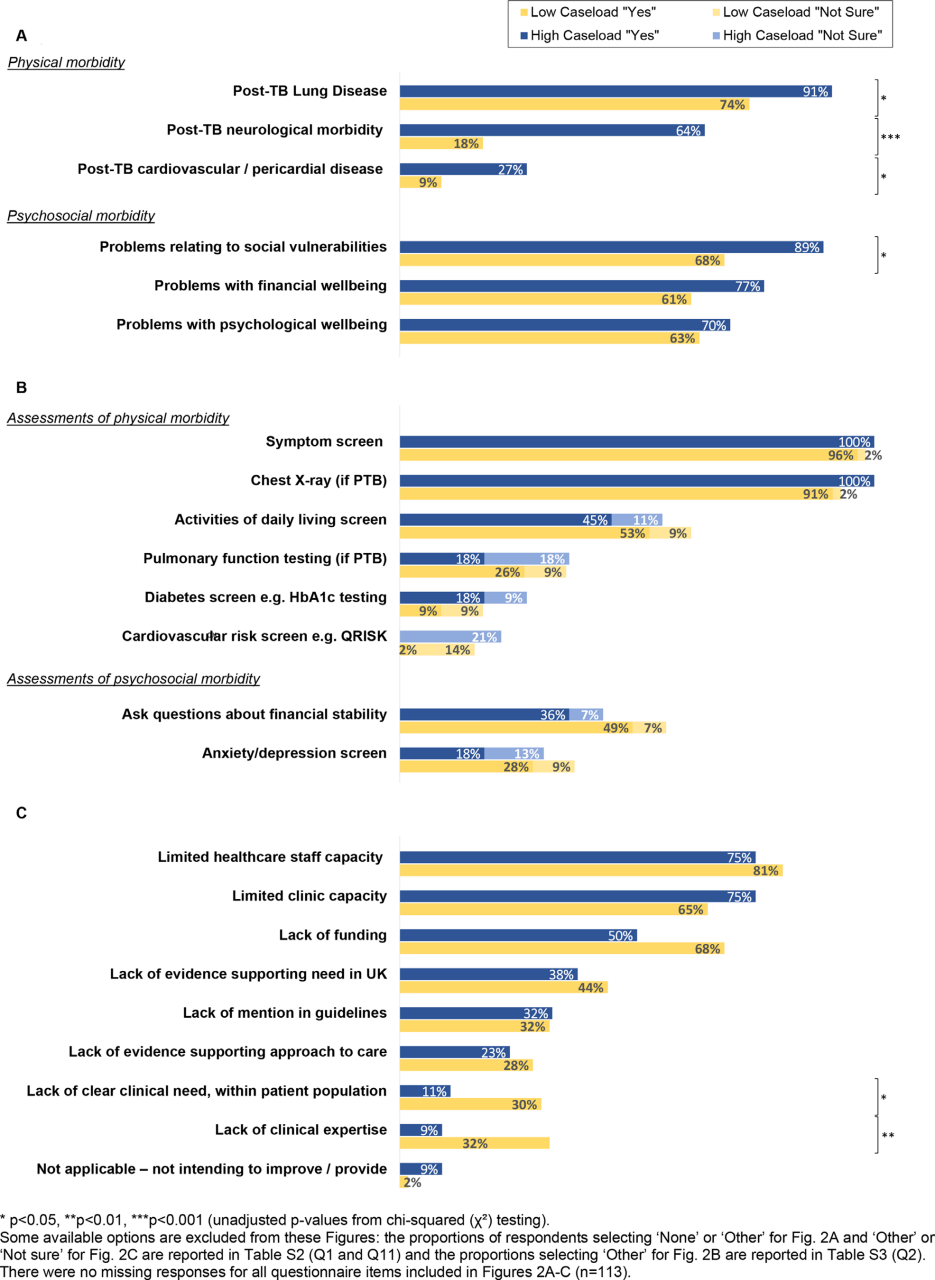
The proportions of survey respondents (n=113) reporting they encounter types of post-TB morbidity (A), perform end of treatment assessments for TB survivors (B), and experience challenges to providing post-TB care (C), by caseload of TB service (low <30, high ≥30 cases/year). HbA1c, haemoglobin A1c; PTB, pulmonary TB; TB, tuberculosis.

Respondents from high caseload services reported seeing a broader range of post-TB morbidity; the mean number of types encountered among high caseload services was 4.2 (SD 1.5) compared with 2.9 (SD 1.5) for low caseload services (p<0.001). Respondents from high caseload services more often selected post-TB neurological morbidity (64% among high vs 18% among low caseload services; p<0.001), cardiovascular and pericardial disease (27% vs 9%; p=0.01), lung disease (91% vs 74%; p=0.02) and problems relating to social vulnerabilities (89% vs 68%); p=0.01) within the survey (figure 2A*,* online supplemental table S2).

### Post-TB follow-up

Most respondents (104/113; 92%) reported at least one reason for following up TBS after treatment. The most common reasons for follow-up included a high TB disease burden at diagnosis (82%; 93/113), concern about incomplete TB treatment (81%; 91/113), the presence of residual lung disease (67%; 76/113) and follow-up after treatment for multidrug-resistant (MDR-) or extensively drug-resistant (XDR-) TB (65%; 74/113). Anxiety or depression (4%; 4/113) and being socioeconomically vulnerable (6%; 7/113) were the least commonly reported reasons for follow-up (online supplemental table S2).

A greater proportion of respondents from high caseload TB services (82%; 46/56) reported following up MDR/XDR TBS after treatment, compared with those from low caseload services (49%; 28/57) (p<0.001) (online supplemental table S2).

### End of TB treatment assessments

At or around the time of TB treatment completion, almost all TB clinicians routinely screen for symptoms (ask questions to check on persisting/new symptoms which may relate to TB) (98%; 111/113) and perform chest X-rays for people who have completed treatment for pulmonary TB (PTB) (96%; 108/113). However, the proportion of respondents reporting routine screening for cardiovascular risk (1%; 1/113) or diabetes (13%; 15/113) at treatment completion was low, and several respondents were unsure whether these services were being offered (20/113 for cardiovascular risk and 10/113 for diabetes screening). Fewer than half reported performing pulmonary function testing for PTB survivors (22%; 25/113), or activities of daily living screens (49%; 55/113) and, again, several were unsure (15/113 and 11/113, respectively) whether these were offered (online supplemental table S3).

Despite the high proportions of respondents recognising psychosocial post-TB morbidity among their patient population, lower proportions reported screening for anxiety and depression (23%; 26/113) and financial instability (42%; 48/113) at or around the time of TB treatment completion (figure 2B*,* online supplemental table S3).

### Provision of post-TB care

Fewer than half (41%; 46/113) of respondents reported that their TB service routinely provides ongoing medical care after anti-TB treatment directly and only 11% (12/113) reported ongoing psychosocial support. However, almost all (92%; 104/113) respondents reported that their TB service routinely provides advice to the patient’s GP and many (71%; 80/113) refer them to another specialty or service at the end of treatment (online supplemental table S3).

Where provided, post-TB care mostly occurs outside dedicated clinics; of the 53 respondents reporting that their TB service provides ‘ongoing medical care,’ ‘psychosocial support’ or ‘another type of direct support,’ only 19 (36%) reported this occurs ‘via a specific post-TB clinic.’ Among these 19, three respondents provided detail (via free text) on when care occurs: they described reviews at 3-months (2 respondents) or 6- and 12-months (1 respondent) post-treatment completion. Around half (51%; 27/53) reported the follow-up occurs at the discretion of individual clinicians and over a third (38%; 20/53) reported that it occurs on an informal or ad hoc basis, for example, by providing a TB nurse advice line number and following up on a patient-initiated basis (free text theme). Around a quarter of respondents (26%; 14/53) reported diverse pathways for post-TB care within their TB service (online supplemental table S2).

Free text entries suggest that follow-up depends on individual patient factors, for example, the complexity of their TB disease, medication-related issues or the nature of any ongoing symptoms.

Advice on aspects of post-TB care is infrequently included in local TB guidelines. Of the 61 respondents confirming that their TB service has a local TB guideline, just over a third (38%; 23/61) reported that it contains any type of advice on activities relating to the care of TBS and the remainder reported it provides no advice (39%; 24/61) or were unsure (23%; 14/61). Where advice on post-TB care was included in the guideline, it mostly focused on ensuring advice was provided to GPs (96%; 22/23) (online supplemental table S2).

### Challenges and future plans

Most respondents (86%; 97/113) identified multiple challenges to providing post-TB care within their TB service, of which the most common were limited healthcare staff (78%; 88/113), limited clinic capacity (70%; 79/113) and funding (59%; 67/113), a lack of evidence (41%; 46/113) and lack of mention in guidelines (32%; 36/113) (figure 2C*,* online supplemental table S2)*.* Only a fifth of respondents reported lack of clinical expertise in post-TB morbidity (20%; 23/113) and lack of clinical need (within their population) (20%; 23/113) as challenges to providing post-TB care.

Challenges around a lack of clinical expertise (33% vs 9% (p=0.002)) and lack of clinical need (30% vs 11% (p=0.01)) were more common in low compared with high caseload services (figure 2D*,* online supplemental table S2).

Only a small proportion (9%; 10/111) of respondents reported that their TB service currently has plans to incorporate activities focused on post-TB morbidity (online supplemental table S3). Among these 10, some (4/10) detailed that such plans involved setting up post-TB clinics, and that these clinics might care for TBS with specific types of TB sequelae, for example, following neurological or spinal TB.

## Discussion

This national survey is the first to describe current approaches to post-TB care in the UK. Both physical and psychosocial post-TB morbidity are widely recognised among TBS, by specialist TB clinicians. However, most TB services experience multiple challenges to providing post-TB care, including resource constraints and the lack of evidence and guidelines. There is variability in the screening assessments and care which TB services provide at, or after, TB treatment completion, with fewer than half reporting routine post-TB medical care or ongoing psychosocial support. We also found differences in the types of post-TB morbidity encountered and provision of care depending on the caseload of the TB service.

TB clinicians in the UK recognise a broad range of patterns of post-TB morbidity among their patients. Most had encountered post-TB lung disease, neurological sequelae and problems relating to social vulnerabilities, financial or psychological well-being. These findings align with data emerging from other countries, which describe the high burden of long-term sequelae among TBS, including after PTB and TB meningitis.^2 9 20 21^ However, data on the prevalence of post-TB morbidity in the UK is limited. One recent retrospective cohort study using national data identified an elevated incidence of cardiovascular events among TBS compared with matched controls (adjusted incidence rate ratio 1.51, during the period from 3 months after diagnosis up to 2 years).^22^ Beyond this, to our knowledge, no UK-based epidemiological studies have characterised the physical or psychosocial outcomes of TBS. Our data indicate that UK clinicians encounter diverse patterns of post-TB sequelae in routine clinical practice. However, this study was not designed to support epidemiological inference. Robust context-specific population-level data are therefore needed to confirm clinicians’ impressions and to inform future strategies for post-TB care.

We also found that clinicians in high caseload TB services reported a broader range of post-TB morbidity compared with those in low caseload settings; a greater proportion reported seeing TBS with neurological, lung and cardiovascular sequelae, as well as problems relating to social vulnerabilities. This may reflect the higher patient numbers in these facilities, the concentration of patients with more complex disease in specialist centres, or increased capacity to consider post-TB sequelae in larger, better-resourced TB facilities. However, we did not adjust for other factors, and more data are needed to accurately determine where long-term care needs are concentrated.

We identified limited screening for post-TB sequelae and comorbidities within UK services. Fewer than a quarter routinely conduct pulmonary function testing for pulmonary TBS. Screening for conditions including diabetes and cardiovascular disease, which have elevated incidence among TBS,^322^,^24^ is even less common. Despite clinicians in the UK frequently recognising ongoing psychosocial morbidity among their patients, formal screening for these problems remains rare. In contrast, nearly all services perform an end-of-treatment evaluation through chest X-ray and symptom review. This suggests that TB care in the UK remains centred on the acute disease episode, rather than long-term morbidity. The importance of TB multimorbidity and post-TB sequelae is increasingly recognised globally,^25^,^27^ and while some countries have incorporated morbidity screening for TBS into their guidelines,^16 17^ most national policies and treatment targets still omit post-TB morbidity.^17 28 29^ This includes many high-income settings—a recent survey from the Netherlands found that only 38% of physicians routinely planned follow-up despite broad recognition of post-TB morbidity.^30^ Further data on the burden of post-TB morbidity in the UK will help to inform the need for routine morbidity screening and care in this setting.

Although post-TB care is not mandated in UK guidelines, many clinicians are nonetheless implementing elements of follow-up. However, post-TB care pathways vary considerably within and between services. Fewer than half provide direct follow-up and where this exists, delivery is inconsistent—relying on a mix of clinician discretion, ad hoc arrangements and dedicated clinics. Such heterogeneity may create disparities in access and quality of care, which disproportionately affect TBS from socially vulnerable groups.^31^ In high caseload services, follow-up appears more often clinician-led, and lack of expertise or perceived clinical need is cited less frequently as a barrier. Regional variation in case-mix (eg, PTB vs extrapulmonary TB)^32^ and the more limited staffing of smaller, lower caseload clinics (fewer multidisciplinary teams and TB nurses)^33^ likely shape familiarity with post-TB conditions and how post-TB care is organised. Across settings, many clinicians also reported referring patients routinely to other services, reflecting efforts to maintain continuity of care. Together, these findings point to a need for more structured guidance to support UK TB services and ensure equitable access.

Experiences from other settings point to several potential models for post-TB care, but the optimal approach in the UK will likely require local adaptation and testing. Practical delivery options may include provision of post-TB care within TB services, for example through end-of-treatment assessment and basic post-TB lung disease screening and rehabilitation,^25 34 35^ onwards linkage from TB services to dedicated multidisciplinary follow-up, analogous to NHS Long-COVID clinics,^36^ or referral of TBS with residual morbidity into existing services (respiratory, primary care and mental health) with clearly defined referral pathways.^25 27^

Most TB services reported multiple challenges in delivering post-TB care. Key barriers include those relating to capacity (limited staff, clinic capacity and funding) as well as the absence of national evidence and guidance. Similar obstacles have been documented in both high- and low-income settings,^2137^,^39^ and understanding them is particularly important for TB services, which care for socially and medically vulnerable populations.^37 40^ Romanowski *et al* emphasise the value of mapping such barriers to feasible solutions when developing post-TB care packages, offering a practical framework that can be adapted to other settings.^38^ Given heterogeneity in caseloads, staffing and service configuration across the UK, pathways should be piloted and adapted to local resources and expertise before wider roll-out.

This national survey provides the first UK-wide snapshot of clinicians’ awareness of post-TB morbidity and current post-TB care. Strengths of the survey include that we invited all UK TB services to participate, achieved a high response rate and received substantial free-text input that added nuance and signalled strong engagement.

Limitations include that several constructs within the survey lack standard definitions—while this allowed for diverse models of practice to be reported by respondents, it does mean that these concepts could have been interpreted differently. Related to this, we had included one question item on funding which was too ambiguous for robust inference and is presented only in online supplemental table S2. We restricted analysis to one response per service and are unable to comment on within-service variation between clinicians. Use of researcher-defined response categories limited granularity, although ‘other’ was rarely selected, suggesting the categories we provided were comprehensive. Multiple comparisons were conducted across caseload strata without formal adjustment, increasing the risk of type I error. Additionally, the survey did not include specific questions on clinicians’ approaches to respiratory services including pulmonary rehabilitation and smoking cessation, which warrants focused evaluation in future studies. Lastly, we recognise that our data are self-reported, and that further service-level and epidemiological data, alongside input from TBS regarding their lived experiences, are needed to understand morbidity and care delivery.

## Conclusions

This national survey shows that UK TB clinicians recognise post-TB morbidity as a challenge for their patients and are already introducing elements of care, but provision is heterogeneous. Further UK-specific data on the burden, patterns and distribution of post-TB morbidity are needed in order to determine the need for post-TB care in the UK. Clear, pragmatic guidance is needed to ensure standardised approaches to care in the UK setting. Engagement with stakeholders including patients, clinicians and policymakers must be used to identify the core components of post-TB care, to clarify the role of TB services in post-TB morbidity screening and care, and to address the barriers identified here. Pilot implementation studies in diverse settings in the UK should assess feasibility, acceptability and equity, informing scalable models. Current variability in care diminishes equity, and our findings suggest a need for a consistent, coordinated and integrated strategy for post-TB care within the UK.

## Supplementary material

10.1136/bmjresp-2025-004021 online supplemental file 1

10.1136/bmjresp-2025-004021 online supplemental file 2

10.1136/bmjresp-2025-004021 online supplemental table 1

## Data Availability

Data are available on reasonable request.
