# Supplementary material for: Post-TB care in the UK: a national survey of existing practice
Source: BMJ Open Respir Res. 2026 Feb 26;13(1):e004021. doi: 10.1136/bmjresp-2025-004021 (PMC12958935; doi:10.1136/bmjresp-2025-004021)
Supplement: online supplemental file 1 [file bmjresp-13-1-s001.pdf]

HOPE-TBS National Survey of Post-TB care

This survey aims to understand the current approaches of TB services to post-TB care.

HOPE-TBS Survey Participant Information Sheet

Thank you for your interest in the HOPE-TBS Survey.

Please click on the attachment below to read the Participant Information Sheet (PIS) for this survey.

Then, please click 'Next Page' below.

[Attachment: "Participant Information Sheet (HOPE-TBS).docx"]

HOPE-TBS Consent Form

If you are happy to proceed, please check that you agree with the statements below before providing your consent to participate.

	Agree	Disagree
I confirm that I have read and understood the Participant Information Sheet for the HOPE-TBS Survey.	○	○
I have been given the opportunity to consider the information provided, ask questions and have had these questions answered to my satisfaction.	○	○
I understand that my participation is voluntary and that I am free to withdraw at any time, without giving any reason, and without my employment or legal rights being affected.	○	○
I understand that my information (including direct quotations from answers I provide in the survey) may be used anonymously in research outputs, including reports and publications. I understand that this information will be deidentified (coded) and I will not be identifiable in any way.	○	○

I understand that relevant sections of my data collected during the study may be looked at by individuals from the University of Leicester (Sponsor), from regulatory authorities, or from my participating NHS Trust, where it is relevant to my taking part in this research. I give permission for these individuals to have access to this data.	◯	◯

I agree to take part in this study.

◯ Yes
◯ No

Thank you for your time but you are not a suitable participant for this survey.

Thank you for agreeing to take part, please click 'Next Page' to proceed.

HOPE-TBS Questionnaire

Please note the following whilst answering the survey questions:

In order for us to gain a broad understanding of current post-TB care, the questions ask about what your TB service as a whole is doing routinely rather than your approach as an individual.

Please feel free to discuss your responses with the rest of your clinical team.

Your answers will be stored as you go along, so you can pause the questionnaire and resume it later if you want. The progress bar at the bottom will display your progress in %.

For the purpose of this survey, questions are based on the following definitions:

TB survivors: People who completed treatment for TB disease, at any point in the past.

Post-TB morbidity: Physical impairments and psychosocial morbidity which start or persist after a person has completed TB treatment, including persisting adverse effects of treatment.

Post-TB care: Includes activities aimed at identifying, preventing or managing post-TB morbidity (including care of mental health).

Questions about your role in the TB service

Please select the region of the UK in which you work.

- ◯ England: East Midlands
- ◯ England: East of England
- ◯ England: London
- ◯ England: North East and Yorkshire
- ◯ England: North West
- ◯ England: South East
- ◯ England: South West
- ◯ England: West Midlands
- ◯ Northern Ireland
- ◯ Scotland
- ◯ Wales

Where do you work, when providing TB care?

- ◯ Chesterfield Royal Hospital NHS Foundation Trust
- ◯ Nottingham University Hospitals NHS Trust
- ◯ Sherwood Forest Hospitals NHS Foundation Trust
- ◯ United Lincolnshire Hospitals NHS Trust
- ◯ University Hospitals of Derby and Burton NHS Foundation Trust
- ◯ University Hospitals of Leicester NHS Trust
- ◯ University Hospitals of Northamptonshire NHS Group
- ◯ Other organisation - not listed here

Where do you work, when providing TB care?

- ◯ Bedfordshire Hospitals NHS Foundation Trust
- ◯ Cambridge University Hospitals NHS Foundation Trust
- ◯ East & North Hertfordshire NHS Trust
- ◯ East Suffolk and North Essex NHS Foundation Trust
- ◯ Essex Partnership University NHS Foundation Trust (EPUT)
- ◯ James Paget University Hospitals NHS Foundation Trust
- ◯ Mid and South Essex NHS Foundation Trust
- ◯ Milton Keynes University Hospital NHS Foundation Trust
- ◯ Norfolk & Norwich University Hospitals NHS Foundation Trust
- ◯ North West Anglia NHS Foundation Trust
- ◯ Princess Alexandra Hospital NHS Trust
- ◯ Queen Elizabeth Hospital King's Lynn NHS Foundation Trust
- ◯ West Hertfordshire Teaching Hospitals NHS Trust
- ◯ West Suffolk NHS Foundation Trust
- ◯ Other organisation - not listed here

Where do you work, when providing TB care?

- ◯ Havering and Redbridge University Hospitals NHS Trust
- ◯ Barts Health NHS Trust
- ◯ Chelsea & Westminster Hospital NHS Foundation Trust
- ◯ Croydon Health Services NHS Trust
- ◯ Epsom and St Helier University Hospitals NHS Trust
- ◯ Guys' and St Thomas' NHS Foundation Trust
- ◯ Homerton Healthcare NHS Foundation Trust
- ◯ Imperial College Healthcare NHS Trust
- ◯ King's College Hospital NHS Foundation Trust
- ◯ Kingston Hospital NHS Foundation Trust
- ◯ Lewisham and Greenwich NHS Trust
- ◯ London North West University Healthcare NHS Trust
- ◯ Oxleas NHS Foundation Trust (Greenwich & Bexley TB service)
- ◯ St George's Hospital
- ◯ The Hillingdon Hospitals NHS Foundation Trust
- ◯ Whittington NHS Trust (North Central London TB Service)
- ◯ Other organisation - not listed here

Where do you work, when providing TB care?

- ◯ Airedale NHS Foundation Trust
- ◯ Bradford Teaching Hospitals NHS Foundation Trust
- ◯ Calderdale and Huddersfield NHS Foundation Trust
- ◯ City Health Care Partnership CIC (Hull and East Riding Clinics)
- ◯ County Durham and Darlington NHS Foundation Trust
- ◯ Doncaster and Bassetlaw Teaching Hospitals NHS Foundation Trust
- ◯ Gateshead Health NHS Foundation Trust
- ◯ Harrogate and District NHS Foundation Trust (North Yorkshire TB Nursing Service)
- ◯ Hull University Teaching Hospitals NHS Trust
- ◯ Leeds Teaching Hospitals NHS Trust
- ◯ Locala Community Partnerships CIC
- ◯ North Tees and Hartlepool NHS Foundation Trust
- ◯ Northern Lincolnshire & Goole NHS Foundation Trust
- ◯ Rotherham NHS Foundation Trust
- ◯ Doncaster and South Humber NHS Foundation Trust
- ◯ Sheffield Teaching Hospitals NHS Foundation Trust
- ◯ South Tees Hospitals NHS Foundation Trust
- ◯ South Tyneside and Sunderland NHS Foundation Trust
- ◯ South West Yorkshire Partnership NHS Foundation Trust
- ◯ The Newcastle upon Tyne Hospitals NHS Foundation Trust (North of Tyne TB Service)
- ◯ York and Scarborough Teaching Hospitals NHS Foundation Trust
- ◯ Other organisation - not listed here

Where do you work, when providing TB care?

- ◯ Bolton NHS Foundation Trust
- ◯ Bridgewater Community Healthcare NHS Foundation Trust
- ◯ Cheshire and Wirral Partnership NHS Foundation Trust
- ◯ East Lancashire Hospitals NHS Trust
- ◯ Lancashire and South Cumbria NHS Foundation Trust
- ◯ Lancashire Teaching Hospitals NHS Foundation Trust
- ◯ Liverpool University Hospitals NHS Foundation Trust
- ◯ Manchester University NHS Foundation Trust (MFT)
- ◯ North Cumbria Integrated Care NHS Foundation Trust
- ◯ Northern Care Alliance NHS Foundation Trust (NCA)
- ◯ Stockport NHS Foundation Trust
- ◯ Tameside and Glossop Integrated Care NHS Foundation Trust
- ◯ University Hospitals of Morecambe Bay NHS Foundation Trust
- ◯ Wirral University Teaching Hospital NHS Foundation Trust
- ◯ Wigan & Leigh Teaching Hospitals NHS Foundation Trust
- ◯ Other organisation - not listed here

Where do you work, when providing TB care?

- ◯ Ashford & St Peter's Hospitals NHS Foundation Trust
- ◯ Buckinghamshire Healthcare NHS Trust
- ◯ East Sussex Healthcare NHS Trust
- ◯ Frimley Health NHS Foundation Trust
- ◯ Hampshire and Isle of Wight Healthcare NHS Foundation Trust
- ◯ Hampshire Hospitals NHS Foundation Trust
- ◯ Kent Community Health NHS Foundation Trust
- ◯ Maidstone and Tunbridge Wells NHS Trust
- ◯ Medway NHS Foundation Trust
- ◯ Oxford University Hospitals NHS Foundation Trust
- ◯ Portsmouth Hospitals University NHS Trust
- ◯ Royal Berkshire NHS Foundation Trust
- ◯ Royal Surrey NHS Foundation Trust
- ◯ St Mary's Hospital (Isle of Wight)
- ◯ Surrey & Sussex Healthcare NHS Trust
- ◯ University Hospitals Sussex NHS Foundation Trust
- ◯ Other organisation - not listed here

Where do you work, when providing TB care?

- ◯ Cornwall Partnership NHS Foundation Trust
- ◯ Dorset County Hospital NHS Foundation Trust
- ◯ Gloucestershire Hospitals NHS Foundation Trust
- ◯ Royal Cornwall Hospitals NHS Trust
- ◯ Royal Devon University Healthcare NHS Foundation Trust
- ◯ Royal United Hospitals Bath NHS Foundation Trust
- ◯ Salisbury NHS Foundation Trust
- ◯ Somerset NHS Foundation Trust
- ◯ Torbay and South Devon NHS Foundation Trust
- ◯ University Hospitals Bristol and Weston NHS Foundation Trust
- ◯ University Hospitals Dorset NHS Foundation Trust
- ◯ University Hospitals Plymouth NHS Trust
- ◯ Other area/organisation - not listed here

Where do you work, when providing TB care?	◯ Sandwell & West Birmingham NHS Trust<br>◯ Shrewsbury & Telford Hospital NHS Trust<br>◯ The Dudley Group NHS Foundation Trust<br>◯ The Royal Wolverhampton NHS Trust<br>◯ University Hospitals Birmingham NHS Foundation Trust<br>◯ University Hospitals Coventry & Warwickshire NHS Trust<br>◯ University Hospitals North Midlands NHS Trust<br>◯ Walsall Healthcare NHS Trust<br>◯ Worcestershire Acute Hospitals NHS Trust<br>◯ Wye Valley NHS Trust<br>◯ Other area/organisation - not listed here
Where do you work, when providing TB care?	◯ Belfast Trust: Musgrave Park Hospital<br>◯ Belfast Trust: Royal Victoria Hospital<br>◯ Northern Trust<br>◯ South Eastern Trust<br>◯ Southern Trust<br>◯ Western Trust<br>◯ Other area/organisation - not listed here
Where do you work, when providing TB care?	◯ Ayrshire and Arran<br>◯ Borders<br>◯ Dumfries & Galloway<br>◯ Fife<br>◯ Forth Valley<br>◯ Grampian<br>◯ Greater Glasgow & Clyde: Cyde Sector<br>◯ Greater Glasgow & Clyde: North Glasgow<br>◯ Highland<br>◯ Lanarkshire<br>◯ Lothian<br>◯ Orkney<br>◯ Shetland<br>◯ Tayside<br>◯ Western Isles<br>◯ Other area/organisation - not listed here
Where do you work, when providing TB care?	◯ Aneurin Bevan UHB<br>◯ Betsi Cadwaladr UHB<br>◯ Cardiff & Vale UHB<br>◯ Cwm Taf Morgannwg UHB<br>◯ Hywel Dda UHB<br>◯ Swansea Bay UHB<br>◯ Other area/organisation - not listed here
If your TB service has not been listed, please enter its name here.	__________________________________
Please select your role.	◯ Respiratory Consultant<br>◯ Infectious Diseases or Microbiology Consultant<br>◯ Specialist TB Nurse<br>◯ Other
Please describe your role.	__________________________________
Are you the Lead Nurse or Lead Clinician?	◯ Yes<br>◯ No

10%

Questions about your TB service's workforce and size

For some of these questions, the same information was collected via the GIRFT Review of TB services and so you may find it helpful to cross-check your answers with your local GIRFT datapack.

How many WTE (Whole Time Equivalent) Specialist TB Nurses (Band 5-8) work in your TB clinic?

- ◯ 0 - 1.0
- ◯ 1.1 - 2.0
- ◯ 2.1 - 3.0
- ◯ 3.1 - 4.0
- ◯ 4.1 - 5.0
- ◯ 5.1 - 6.0
- ◯ 6.1 - 7.0
- ◯ 7.1 - 8.0
- ◯ More than 8
- ◯ Not sure

How many Consultants who specialise in TB work in your TB clinic in total?

____
(Please enter an integer.)

Are you certain that your TB service includes 10 or more consultants?

- ◯ Yes
- ◯ No

Please re-enter the correct number of consultants in the box for the question above, when you are ready.

OK, please move on to the next question.

What was the estimated caseload (number of notified active TB cases in 1 year) for your service in 2023?

You may give a rough estimate.

Or, if you are not sure, you may leave blank and move on to the next question.

____
(Please enter an integer.)

20%

Question about nature of post-TB morbidity

Based on your experience within your TB service, which of the following have you come across among TB survivors?	☐ Post-TB lung disease<br>☐ Post-TB cardiovascular and pericardial disease<br>☐ Post-TB neurological morbidity<br>☐ Problems with financial wellbeing<br>☐ Problems with psychological wellbeing<br>☐ Problems relating to social vulnerabilities such as homelessness, drug or alcohol dependency or forced migrant status<br>☐ None of the above<br>☐ Other - please specify<br>(You may select more than one option.)
Please specify these other types of issues.	______________________________

30%

AT OR AROUND THE TIME OF TB TREATMENT COMPLETION, within your TB service, do clinicians routinely perform any of the following for TB survivors?

	Yes	No	Not sure
Symptom screen - ask questions to check on persisting / new symptoms which may relate to TB	○	○	○
Activities of daily living screen - ask questions to check whether day-to-day self-care tasks have become more difficult	○	○	○
Anxiety/depression screen	○	○	○
Cardiovascular risk screen e.g. using QRISK score	○	○	○
Ask questions about financial stability e.g. issues with job security, housing and benefits	○	○	○
Request pulmonary function testing (in the context of pulmonary TB)	○	○	○
Request Chest X-ray (in the context of pulmonary TB)	○	○	○
Diabetes screening e.g. HbA1c testing	○	○	○
Other assessments or investigations not listed here	○	○	○

Please specify these other assessments or investigations. ______________________________

50%

AT OR AROUND THE TIME OF TB TREATMENT COMPLETION, does your TB service currently routinely provide any of the following for TB survivors?

	Yes	No	Not sure
Onward referral to appropriate specialty or service	○	○	○
Advice to GP	○	○	○

60%

AFTER TB TREATMENT COMPLETION, does your TB service currently routinely provide any of the following for TB survivors?

	Yes	No	Not sure
Direct provision of ongoing medical care	○	○	○
Direct provision of ongoing psychosocial support	○	○	○
Another type of direct support	○	○	○

Please specify any other direct support provided.

____

How is this post-TB care provided?

- ☐ Via a specific post-TB clinic
- ☐ On an informal or ad hoc basis
- ☐ At the discretion of the individual clinician
- ☐ Other

(You may select multiple options.)

If you wish, please specify details.

____

Within your TB service, which TB survivors do you routinely follow up, after TB treatment completion?

- ☐ Treated for MDR/XDR -TB
- ☐ High TB disease burden, at diagnosis (e.g. complex or disseminated disease)
- ☐ Concern relating to incomplete TB treatment (e.g. treatment stopped early or there were adherence challenges)
- ☐ Residual drug side effects
- ☐ Residual lung disease
- ☐ Residual functional and/or musculoskeletal impairment
- ☐ Anxiety or depression
- ☐ Socio-economically vulnerable
- ☐ Concern about nutritional status or weight gain
- ☐ None of the above (no follow up)
- ☐ Other reasons for follow up

(Please select any which apply.)

Please specify any other reasons for follow up which were not listed above.

____

1. When providing follow up care, how is this funded?

- ☐ The TB service is not providing any form of follow up care
- ☐ Unfunded - all follow up care is being provided informally
- ☐ National funding through NHS England
- ☐ Local ICB (Integrated Care Board)
- ☐ NHS Hospital Trust
- ☐ Research funding
- ☐ Other

(Please select any which apply)

Please specify which other type of funding is being received.

____

Does your TB service have a local TB guideline?	◯ Yes<br>◯ No<br>◯ Don't know
Does this local TB guideline provide advice on any of the following activities relating to the care of TB survivors?	☐ Screening for residual physical morbidity caused by TB disease<br>☐ Screening for residual psychological morbidity related to the TB episode<br>☐ Linkage to care with other providers<br>☐ Advice to GP<br>☐ Direct provision of post-TB medical care<br>☐ Direct provision of post-TB psychosocial support<br>☐ Other post-TB activities<br>☐ None of the above<br>☐ Not sure<br>(Please select any which apply.)
Please specify	__________________________________

80%

Future plans and current challenges

Does your TB service currently have any future plans to incorporate activities focused on post-TB morbidity?

- ◯ Yes
- ◯ No
- ◯ Not sure

Please could you specify these future plans if possible.

____

What are the main challenges for your TB service in providing more comprehensive post-TB care?

- ☐ Not applicable - not intending to provide post-TB care
- ☐ Lack of funding
- ☐ Limited healthcare staff capacity
- ☐ Limited clinic capacity e.g. space or availability of appointments
- ☐ Lack of mention in guidelines
- ☐ Lack of evidence supporting the need for post-TB services in the UK
- ☐ Lack of evidence supporting the approach to care for post-TB morbidity
- ☐ Lack of clinical expertise to manage post-TB morbidities
- ☐ Lack of clear clinical need, within our patient population
- ☐ Other
- ☐ Not sure

(Please select any which apply.)

Please specify

____

If you would like to, please provide any comments or additional information relating to the topic of post-TB care.

____

90%

Further Research (final question)

If you wish to participate in any further research related to post-TB care, please provide your consent for this below. This may include participating in focus groups for example.

If you do not wish to provide your consent for this, and have completed the survey, please just click Submit below.

Please tick here if you agree to be involved in further research.	☐ I agree to be re-contacted about other ethically approved research related to post-TB care. I understand that I will have the option to consent or decline participation in any further research based on the information provided to me at that time. ((optional))
Please enter your email address here.	______________________________

Thank you.
If you have completed the survey, please now click Submit below.

100%
