# Supplementary material for: Post-TB care in the UK: a national survey of existing practice
Source: BMJ Open Respir Res. 2026 Feb 26;13(1):e004021. doi: 10.1136/bmjresp-2025-004021 (PMC12958935; doi:10.1136/bmjresp-2025-004021)
Supplement: online supplemental file 2 [file bmjresp-13-1-s002.docx]

**Supplement Full Methods: Post-TB care in the UK: A national survey of existing practice**

**Authors:**

A O’Reilly^1,2,3^, C A Martin^1,2,3,4^, S E Cox^5,6^, P Haldar^3,7,8^, D Zenner*^9^, M Pareek*^1,2,3,4^, J Meghji*^10^

*Joint senior authors

**Corresponding author:** Dr Jamilah Meghji, National Heart and Lung Institute, VPD building, Hammersmith Hospital Campus, Du Cain Road, London, W12 0NN, UK.

[j.meghji@imperial.ac.uk](mailto:j.meghji@imperial.ac.uk)

Tel: +44 020 7594 8162

**Affiliations:**

1. Division of Public Health and Epidemiology, School of Medical Sciences, College of Life Sciences, University of Leicester, UK
2. Development Centre for Population Health, University of Leicester, Leicester, UK
3. NIHR Leicester Biomedical Research Centre (BRC), University of Leicester, Leicester, UK
4. Department of Infection and HIV Medicine, University Hospitals of Leicester NHS Trust, Leicester, UK
5. National TB Unit, United Kingdom Health Security Agency, London, UK
6. London School of Hygiene and Tropical Medicine, London, UK
7. Division of Respiratory Sciences, School of Medical Sciences, College of Life Sciences, University of Leicester, UK
8. Department of Respiratory Medicine, University Hospitals of Leicester NHS Trust, Leicester, UK
9. Global Public Health Unit, Wolfson Institute of Population Health, Queen Mary University of London, London, UK
10. National Heart and Lung Institute, Imperial College London, London, UK

**Full Methods S1:**

Please note: all sections of **METHODS** in the main manuscript are repeated here, with an additional paragraph within each of the sections entitled *‘Study questionnaire,’ ‘Survey frame,’ Survey distribution and administration’* and *‘Analysis.’*

**METHODS**

We report this study as per the Checklist for Reporting Results of Internet E-Surveys (CHERRIES) (*Checklist S1*).^1^

***Study questionnaire***

We developed and piloted an 11-point digital questionnaire amongst specialist doctors and nurses working in TB clinics (*Questionnaire S1*). The questionnaire was prefaced by definitions for TB survivors, post-TB morbidity and post-TB care, to ensure clarity of these concepts. We collected data on types of post-TB morbidity encountered by TB clinicians; the assessments, investigations and referrals usually performed at the end of TB treatment; and whether any post-TB care is currently being provided or planned by the service. For most questionnaire items, respondents were asked to either select from a list of options provided (including ‘other’ and ‘none’), or to select from the options ‘Yes,’ ‘No’ or ‘Not sure.’ For some questions, respondents could enter free text answers.

We also collected descriptive data on whether a respondent was the Lead Consultant or Lead nurse for their service, their specialty and their TB facility’s caseload.

Branching logic was used to reduce the number and complexity of questions. Five of the eleven core questions were mandatory and we have reported which. There was no ‘completeness check’ at the end of the questionnaire although respondents could not submit the survey without answering the mandatory questions. Only submitted questionnaires were extracted for analysis.

***Survey frame***

The survey frame included TB services providing adult care within NHS Trusts (England), University Health Boards (UHBs, Wales), Health and Social Care (HSC) Trusts (Northern Ireland) and Scottish Health Boards. It was based on primary lists of TB services obtained from key stakeholders in each of the four nations (*Acknowledgements*), which we refined with input from regional representatives and study participants.

Regions lacking formal TB services due to low case numbers were not included in the survey frame. These included Orkney, Shetland and Western Isles (Scotland) and Powys (Wales).

The final survey frame included 135 TB services, including 110 in England, 6 in Wales, 13 in Scotland and 6 in Northern Ireland.

***Survey distribution and administration***

We distributed surveys by email between February and May 2025. For England, Wales and Northern Ireland, the survey was sent directly to at least one specialist consultant or nurse per TB service. For Scotland, Public Health Scotland administrators disseminated the survey directly to the Scottish TB Network mailing list.

We gave presentations in regional and national TB meetings (n=12) to encourage participation. Up to three reminder emails and a phone call were used to follow up with services not responding to the initial email invitation.

Survey completion was voluntary and no incentives were offered for participation. All respondents provided written informed electronic consent.

We collected survey data in the RedCAP (Research Electronic Data Capture) survey platform.^2^ One response was analysed per TB service; where multiple responses were submitted, we selected the first response from the most senior clinician*.*

The following stepwise method was used to exclude multiple responses per TB service:

1. TB service leads’ responses were selected preferentially to a non-leads’ responses.
2. If more than one response was from a lead clinician, the most senior clinician’s response was selected preferentially.
3. If responses were from clinicians of the same lead status and seniority level, the first one submitted (chronologically) was selected.

***Analysis***

We analysed numeric data from questionnaire items descriptively. The median self-reported caseload for TB services was used as a pre-specified cut-off (low <median, high ≥median cases/year) for creating a binary caseload variable, by which questionnaire data were stratified. Where caseload was not self-reported, CCG-level data for the TB service was substituted.

Where caseload was not reported we used the average annual number of notifications (2021-2023) for the relevant NHS Clinical commissioning group (CCG) provided in Supplementary Table 7 of the ‘Tuberculosis in England 2024 Report.’ ^3^

We summarised categorical variables using frequency and percentage, and continuous or discrete variables using means (standard deviation [SD]) if normally distributed and medians (interquartile range [IQR]) for other distributions.

We compared responses in low versus high caseload services using chi-squared tests and t-tests for categorical and continuous data respectively. We reported the proportion of missing data for each questionnaire item.

We conducted all numerical analyses using Stata (StataCorp. 2023. *Stata Statistical Software: Release 18*. College Station, TX: StataCorp LLC.) and identified broad themes from the free text responses.

***Ethical approval***

The study was approved by the West of Scotland NHS Research Ethics Committee (REC reference: 24/WS/0151).

***Patient and public involvement***

A study-specific patient and public involvement and engagement (PPIE) group, including TB survivors and specialist TB clinicians, reviewed and refined draft survey questions, advising on wording and acceptability. Their input informed revisions before piloting.

***References*** *(specific to Full Methods)*

1. Eysenbach, G. Improving the Quality of Web Surveys: The Checklist for Reporting Results of Internet E-Surveys (CHERRIES). *J. Med. Internet Res.* **6**, e132 (2004).

2. Harris, P. A. *et al.* The REDCap consortium: Building an international community of software platform partners. *J. Biomed. Inform.* **95**, 103208 (2019).

3. Tuberculosis in England, 2024 report. *GOV.UK* https://www.gov.uk/government/publications/tuberculosis-in-england-2024-report.
