# Supplementary material for: Post-TB care in the UK: a national survey of existing practice
Source: BMJ Open Respir Res. 2026 Feb 26;13(1):e004021. doi: 10.1136/bmjresp-2025-004021 (PMC12958935; doi:10.1136/bmjresp-2025-004021)
Supplement: online supplemental table 1 [file bmjresp-13-1-s003.pdf]

Table S1: Description of participating TB clinicians and TB services represented (n=113)

Type of TB clinician completing questionnaire	Number (%)
Specialist nurse	73 (65%)
TB nurse	70 (62%)
Respiratory nurse	1 (0.9%)
Health Protection nurse with TB remit	2 (1.8%)
Lead nurse	56 (50%)
Non-lead nurse	17 (15%)
Specialist doctor	40 (35%)
Consultant in Respiratory Medicine	33 (29%)
Consultant in Infectious Diseases or Microbiology	7 (6%)
Lead Consultant	35 (31%)
Non-lead Consultant	5 (4%)

Characteristics of TB services represented	Median (IQR)
Caseload (self-reported number of active TB cases in 2023)	30 (15-61)*
Number of Whole Time Equivalent (WTE) nurses	1.1-2.0 (1.1-4.0)
Number of Consultant doctors	2 (1-3)

*Missing data for self-reported caseload for three TB services. No data missing for any other descriptive category.

Table S2: Proportions of respondents selecting each checklist response, by TB service caseload

**Survey question** *(question type)*	**Total (n = 113)**	**Low caseload, < 30 per year (n = 57)**	**High caseload, ≥ 30 per year (n = 56)**	
**Q1: Based on your experience within your TB service, which of the following have you come across among TB survivors?** *(mandatory to select at least one option)*	**Total (N = 113)**	**Low caseload (N = 57)**	**High caseload (N = 56)**	**High vs. low caseload p-value***
Post-TB Lung Disease	93 (82.3%)	42 (73.7%)	51 (91.1%)	0.02
Post-TB cardiovascular and pericardial disease	20 (17.7%)	5 (8.8%)	15 (26.8%)	0.01
Post-TB neurological morbidity	46 (40.7%)	10 (17.5%)	36 (64.3%)	<0.001
Problems with financial wellbeing	78 (69.0%)	35 (61.4%)	43 (76.8%)	0.08
Problems with psychological wellbeing	75 (66.4%)	36 (63.2%)	39 (69.6%)	0.5
Problems relating to social vulnerabilities, such as homelessness, drug or alcohol dependency or forced migrant status	89 (78.8%)	39 (68.4%)	50 (89.3%)	0.01
None of the above	2 (1.8%)	0 (0.0%)	2 (3.6%)	0.1
Other	5 (4.4%)	5 (8.8%)	0 (0.0%)	0.02
**Q5: How is (this) TB care provided?** *(conditional question for respondents reporting in Q4 they routinely provide direct support (n=53); optional question, multiple options allowed)*	**Total (N = 53)**	**Low caseload (N = 27)**	**High caseload (N = 26)**	**High vs. low caseload p-value***
Via a specific post-TB clinic	19 (35.8%)	12 (44.4%)	7 (26.9%)	0.2
On an informal or ad hoc basis	20 (37.7%)	12 (44.4%)	8 (30.8%)	0.3
At the discretion of the individual clinician	27 (50.9%)	6 (22.2%)	21 (80.8%)	<0.001
Other	2 (3.8%)	1 (3.7%)	1 (3.8%)	1.0

**Q6: Within your TB service, which TB survivors do you routinely follow up, after TB treatment completion?** *(optional, multiple options allowed)*	**Total (N = 113)**	**Low caseload (N = 57)**	**High caseload (N = 56)**	**High vs. low caseload p-value***
Treated for MDR/XDR TB	74 (65.5%)	28 (49.1%)	46 (82.1%)	<0.001
High TB disease burden at diagnosis (e.g. complex or disseminated disease)	93 (82.3%)	46 (80.7%)	47 (83.9%)	0.7
Concern relating to incomplete TB treatment	91 (80.5%)	46 (80.7%)	45 (80.4%)	1.0
Residual drug side effects	52 (46.0%)	26 (45.6%)	26 (46.4%)	0.9
Residual lung disease	76 (67.3%)	33 (57.9%)	43 (76.8%)	0.03
Residual functional and/or musculoskeletal impairment	25 (22.1%)	12 (21.1%)	13 (23.2%)	0.8
Anxiety or depression	4 (3.5%)	3 (5.3%)	1 (1.8%)	0.3
Socio-economically vulnerable	7 (6.2%)	5 (8.8%)	2 (3.6%)	0.3
Concern about nutrition or weight gain	26 (23.0%)	13 (22.8%)	13 (23.2%)	1.0
None of the above (no follow up)	9 (8.0%)	7 (12.3%)	2 (3.6%)	0.09
Other reasons for follow up	6 (5.3%)	3 (5.3%)	3 (5.4%)	1.0

**Q7: When providing follow up care, how is this funded?** *(optional, multiple options allowed)*	**Total (N = 110)**	**Low caseload (N=54)**	**High caseload (N=56)**	**High vs. low caseload p-value***
The TB service is not providing any form of follow up care	7 (6.4%)	4 (7.4%)	3 (5.4%)	0.7
Unfunded - all follow up care is being provided informally	48 (43.6%)	27 (50.0%)	21 (37.5%)	0.2
National funding through NHS England	4 (3.6%)	1 (1.9%)	3 (5.4%)	0.3
Local ICB (Integrated Care Board)	9 (8.2%)	3 (5.6%)	6 (10.7%)	0.3
NHS Hospital Trust	50 (45.5%)	19 (35.2%)	31 (55.4%)	0.03
Research funding	0 (0%)	0 (0%)	0 (0%)	NA
Other	6 (5.5%)	4 (7.4%)	2 (3.6%)	0.4

**Q9: Does the local TB guideline provide advice on any of the following activities relating to the care of TB survivors?** *(conditional question for respondents reporting (in Q8) they have a local TB guideline (n=61); optional, multiple options allowed)*	**Total (N = 61)**	**Low caseload (N=30)**	**High caseload (N=31)**	**High vs. low caseload p-value***
At TB treatment completion:				
Screening for residual physical morbidity caused by TB disease	7 (11.5%)	4 (13.3%)	3 (9.7%)	0.7
Screening for residual psychological morbidity related to the TB episode	2 (3.3%)	2 (6.7%)	0 (0.0%)	0.1
Linkage to care with other providers	8 (13.1%)	6 (20.0%)	2 (6.5%)	0.1
Advice to GP	22 (36.1%)	13 (43.3%)	9 (29.0%)	0.2
After TB treatment completion:				
Direct provision of ongoing medical care	7 (11.5%)	6 (20.0%)	1 (3.2%)	0.04
Direct provision of ongoing psychosocial support	0 (0.0%)	0 (0.0%)	0 (0.0%)	NA
Other activities	2 (3.3%)	2 (6.7%)	0 (0.0%)	0.1
None of the above	24 (39.3%)	12 (40.0%)	12 (38.7%)	0.9
Not sure	14 (23.0%)	4 (13.3%)	10 (32.3%)	0.1

**Q11: What are the main challenges for your TB service in providing more comprehensive post-TB care?** *(optional, multiple options allowed)*	**Total (N = 113)**	**Low caseload (N = 57)**	**High caseload (N = 56)**	**High vs. low caseload p-value***
Not applicable – not intending to improve or provide post-TB care	6 (5.3%)	1 (1.8%)	5 (8.9%)	0.1
Lack of funding	67 (59.3%)	39 (68.4%)	28 (50.0%)	0.05
Limited healthcare staff capacity	88 (77.9%)	46 (80.7%)	42 (75.0%)	0.5
Limited clinic capacity e.g. space or availability of appointments	79 (69.9%)	37 (64.9%)	42 (75.0%)	0.2
Lack of mention in guidelines	36 (31.9%)	18 (31.6%)	18 (32.1%)	0.9
Lack of evidence supporting the need for post-TB services in the UK	46 (40.7%)	25 (43.9%)	21 (37.5%)	0.5
Lack of evidence supporting the approach to care for post-TB morbidity	29 (25.7%)	16 (28.1%)	13 (23.2%)	0.6
Lack of clinical expertise to manage post-TB morbidities	23 (20.4%)	18 (31.6%)	5 (8.9%)	0.003
Lack of clear clinical need, within our patient population	23 (20.4%)	17 (29.8%)	6 (10.7%)	0.01
Other	0 (0.0%)	0 (0.0%)	0 (0.0%)	NA
Not sure	4 (3.5%)	2 (3.5%)	2 (3.6%)	1.0

N = Number of respondents completing the question specified. Proportion of data missing was 0% for all questions other than Q7 which had 2.7% (3/113) missing.
*p-value from chi-squared (χ^2) testing.

Table S3: Proportions of respondents selecting 'Yes,' 'No' or 'Not sure,' by TB service caseload

**Survey question** *(question type)*	**Total (n = 113)**			**Low caseload, < 30 per year (n = 57)**			**High caseload, ≥ 30 per year (n = 56)**			
**Q2: AT OR AROUND THE TIME OF TREATMENT COMPLETION, within your TB service, do clinicians routinely perform any of the following for TB survivors?** *(mandatory question)*	**Total (N=113)**			**Low caseload (N=57)**			**High caseload (N=56)**			**p-value***
	**Yes**	**No**	**Not sure**	**Yes**	**No**	**Not sure**	**Yes**	**No**	**Not sure**	
Symptom screen (ask questions to check on persisting/new symptoms which may relate to TB)	111 (98.2%)	1 (0.9%)	1 (0.9%)	55 (96.5%)	1 (1.8%)	1 (1.8%)	56 (100.0%)	0 (0.0%)	0 (0.0%)	0.4
Chest X-ray (if PTB)	108 (95.6%)	4 (3.5%)	1 (0.9%)	52 (91.2%)	4 (7.0%)	1 (1.8%)	56 (100.0%)	0 (0.0%)	0 (0.0%)	0.1
Activities of daily living screen	55 (48.7%)	47 (41.6%)	11 (9.7%)	30 (52.6%)	22 (38.6%)	5 (8.8%)	25 (44.6%)	25 (44.6%)	6 (10.7%)	0.7
Pulmonary function testing (if PTB)	25 (22.1%)	73 (64.6%)	15 (13.3%)	15 (26.3%)	37 (64.9%)	5 (8.8%)	10 (17.9%)	36 (64.3%)	10 (17.9%)	0.3
Diabetes screen e.g. HbA1c testing	15 (13.3%)	88 (77.9%)	10 (8.8%)	5 (8.8%)	47 (82.5%)	5 (8.8%)	10 (17.9%)	41 (73.2%)	5 (8.9%)	0.4
Cardiovascular risk screen e.g. QRISK	1 (0.9%)	92 (81.4%)	20 (17.7%)	1 (1.8%)	48 (84.2%)	8 (14.0%)	0 (0.0%)	44 (78.6%)	12 (21.4%)	0.4
Anxiety/depression screen	26 (23.0%)	75 (66.4%)	12 (10.6%)	16 (28.1%)	36 (63.2%)	5 (8.8%)	10 (17.9%)	39 (69.6%)	7 (12.5%)	0.4
Ask questions about financial stability	48 (42.5%)	57 (50.4%)	8 (7.1%)	28 (49.1%)	25 (43.9%)	4 (7.0%)	20 (35.7%)	32 (57.1%)	4 (7.1%)	0.3
Other assessments or investigations not listed†	20 (17.7%)	68 (60.2%)	25 (22.1%)	9 (15.8%)	34 (59.6%)	14 (24.6%)	11 (19.6%)	34 (60.7%)	11 (19.6%)	0.8
**Q3: AT OR AROUND THE TIME OF TREATMENT COMPLETION, does your TB service currently routinely provide any of the following?** *(mandatory question)*	**Total (N=113)**			**Low caseload (N=57)**			**High caseload (N=56)**			
	**Yes**	**No**	**Not sure**	**Yes**	**No**	**Not sure**	**Yes**	**No**	**Not sure**	
Onward referral to appropriate specialty or service	80 (70.8%)	26 (23.0%)	7 (6.2%)	38 (66.7%)	15 (26.3%)	4 (7.0%)	42 (75.0%)	11 (19.6%)	3 (5.4%)	0.6
Advice to GP	104 (92.0%)	7 (6.2%)	2 (1.8%)	52 (91.2%)	4 (7.0%)	1 (1.8%)	52 (92.9%)	3 (5.4%)	1 (1.8%)	0.9
**Q4: AFTER TB TREATMENT COMPLETION, does your TB service currently routinely provide any of the following for TB survivors?** *(optional question)*	**Total (N=113)**			**Low caseload (N=57)**			**High caseload (N=56)**			
	**Yes**	**No**	**Not sure**	**Yes**	**No**	**Not sure**	**Yes**	**No**	**Not sure**	
Direct provision of ongoing medical care	46 (40.7%)	60 (53.1%)	7 (6.2%)	22 (38.6%)	30 (52.6%)	5 (8.8%)	24 (42.9%)	30 (53.6%)	2 (3.6%)	0.5
Direct provision of ongoing psychosocial support	12 (10.6%)	94 (83.2%)	7 (6.2%)	8 (14.0%)	46 (80.7%)	3 (5.3%)	4 (7.1%)	48 (85.7%)	4 (7.1%)	0.5
Another type of direct support‡	8 (7.1%)	87 (77.0%)	18 (15.9%)	4 (7.0%)	43 (75.4%)	10 (17.5%)	4 (7.1%)	44 (78.6%)	8 (14.3%)	0.9

**Q10: Does your TB service currently have any future plans to incorporate activities focused on post-TB morbidity?** *(optional question)*	**Total (N=111)**			**Low caseload (N=56)**			**High caseload (N=55)**			**p-value***
	**Yes**	**No**	**Not sure**	**Yes**	**No**	**Not sure**	**Yes**	**No**	**Not sure**	
	10 (9.0%)	60 (54.1%)	41 (36.9%)	2 (3.6%)	34 (60.7%)	20 (35.7%)	8 (14.5%)	26 (47.3%)	21 (38.2%)	0.1

N = Number of respondents completing the question specified. Proportion of data missing was 0% for all questions other than Q10 which had 1.8% (2/113) missing.
*p-value from chi-squared (χ^2) testing.
† 19 of the 20 respondents selecting 'other assessments' specified these via free text, and 13/19 mentioned cross-sectional imaging including CT and/or MRI scans.
‡All 8 selecting 'another type of direct support' specified via free text, and 4/8 described providing access to further advice via TB nurses and/or a telephone line.
